# Echocardiographic evaluation in patient candidate for liver transplant: from pathophysiology to hemodynamic optimization

**DOI:** 10.1186/s44158-024-00211-0

**Published:** 2024-11-14

**Authors:** Marta Iaconi, Micaela Maritti, Giuseppe Maria Ettorre, Luigi Tritapepe

**Affiliations:** 1 Department of Anesthesia and ICU, San Camillo-Forlanini Hospital, Rome, Italy; 2 Surgical Department of Transplants, San Camillo-Forlanini Hospital, Rome, Italy; 3 Anesthesia and ICU, Sapienza University of Rome, Rome, Italy

**Keywords:** Liver transplantation, Echocardiography, Coronary artery disease, Strain, Portopulmonary hypertension, Cirrhosis, Portal hypertension, Intrahepatic vascular resistances, Cirrhotic cardiomyopathy, Diastolic dysfunction, Systolic dysfunction, Hepatopulmonary syndrome

## Abstract

Cardiovascular complications are common in patients with severe liver disease and are an important cause of peri-operative and post-transplant morbidity and mortality. Cirrhotic cardiomyopathy (CCM), often found in advanced liver disease, is characterized by diastolic dysfunction, systolic dysfunction, and electrophysiological abnormalities. While CCM may not cause symptoms at rest, it can become evident during stressful activities, such as surgery. Liver transplantation, while being the definitive treatment for end-stage liver disease (ESLD), carries significant cardiovascular risks. Preoperative cardiac evaluation is essential for assessing these risks and planning appropriate management. Cardiac imaging, particularly echocardiography, plays a crucial role in evaluating liver transplant candidates, helping to identify conditions such as CCM, pulmonary hypertension, hepatopulmonary syndrome, and others. Currently, liver transplant anesthetists must acquire echocardiographic knowledge and skills to evaluate the cardiocirculatory conditions of the transplanted patient, especially in the pre-operative phase, but also intra-operatively and post-operatively.

## Background

Cirrhosis can be caused by various factors including alcohol abuse, hepatitis, non-alcoholic steatohepatitis (NASH), autoimmune conditions, or rarer diseases such as Wilson’s disease, and cryptogenic cirrhosis [1]. It results from hepatic inflammation, fibrosis, angiogenesis, and cell loss. This leads to increased hepatic resistance and portal hypertension, causing the most complications and mortality in patients with cirrhosis [2].

Patients with cirrhosis may experience no symptoms initially, but as the disease progresses, vital organs become compromised due to functional mismatch. Cardiovascular abnormalities, including myocardial dysfunction, are common in cirrhosis, affecting approximately 50% of patients [2]. Patients with cirrhosis often experience increased cardiac output (CO), stroke volume, and decreased systemic vascular resistance (SVR). This hyperdynamic state results from impaired liver function, portal hypertension, and splanchnic vasodilation. Cirrhotic cardiomyopathy (CCM) is a condition characterized by both impaired systolic and diastolic heart function, as well as abnormal electrocardiogram (ECG) readings, particularly in patients with end-stage liver disease (ESLD) [1, 3–5] CCM often remains asymptomatic at rest, manifesting only under stressful conditions despite increased cardiac output. This altered hemodynamic response to stress, in the absence of underlying heart disease, is a hallmark of CCM [5].

Cardiovascular diseases have become more prevalent among liver transplant recipients due to factors like NASH, aging populations, and lifestyle factors (diabetes, obesity, and alcohol abuse). Identifying cardiac problems in these patients can be challenging due to reduced afterload and beta-blocker therapy. Careful selection of liver transplant candidates is crucial, and non-invasive cardiac imaging is increasingly used for pre-operative assessment [6].

**Fig. 1:**
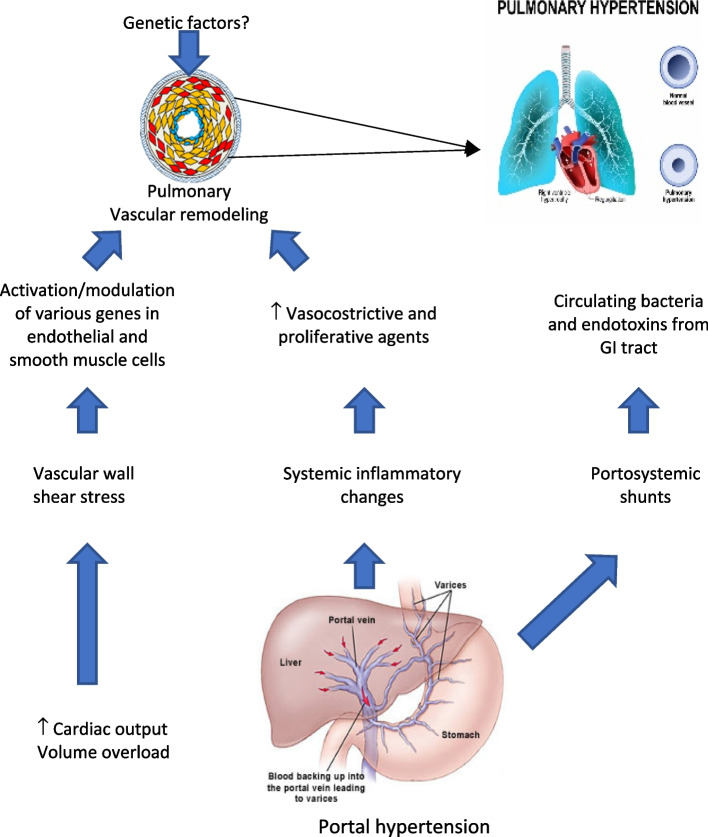
Mechanism of portopulmonary hypertension

Cardiovascular disease remains a significant cause of morbidity and mortality in solid organ transplant recipients, particularly in liver transplantation (LT) [6]. While LT is the definitive treatment for ESLD, CCM associated with it poses challenges for healthcare professionals [7]. Echocardiography is a valuable tool for diagnosing CCM, cardiovascular disease, and other conditions like portopulmonary hypertension (PoPH) and hepatopulmonary syndrome (HPS) [5] (Fig. 1).

Assessing the presence of CCM, PoPH, and HPS is crucial to mitigating their potential negative effects on patient outcomes. Echocardiographic evaluation can be a valuable tool for diagnosing these conditions, particularly prior to LT [2, 5].

## Cirrhosis and hemodynamic disturbances

**Fig. 2:**
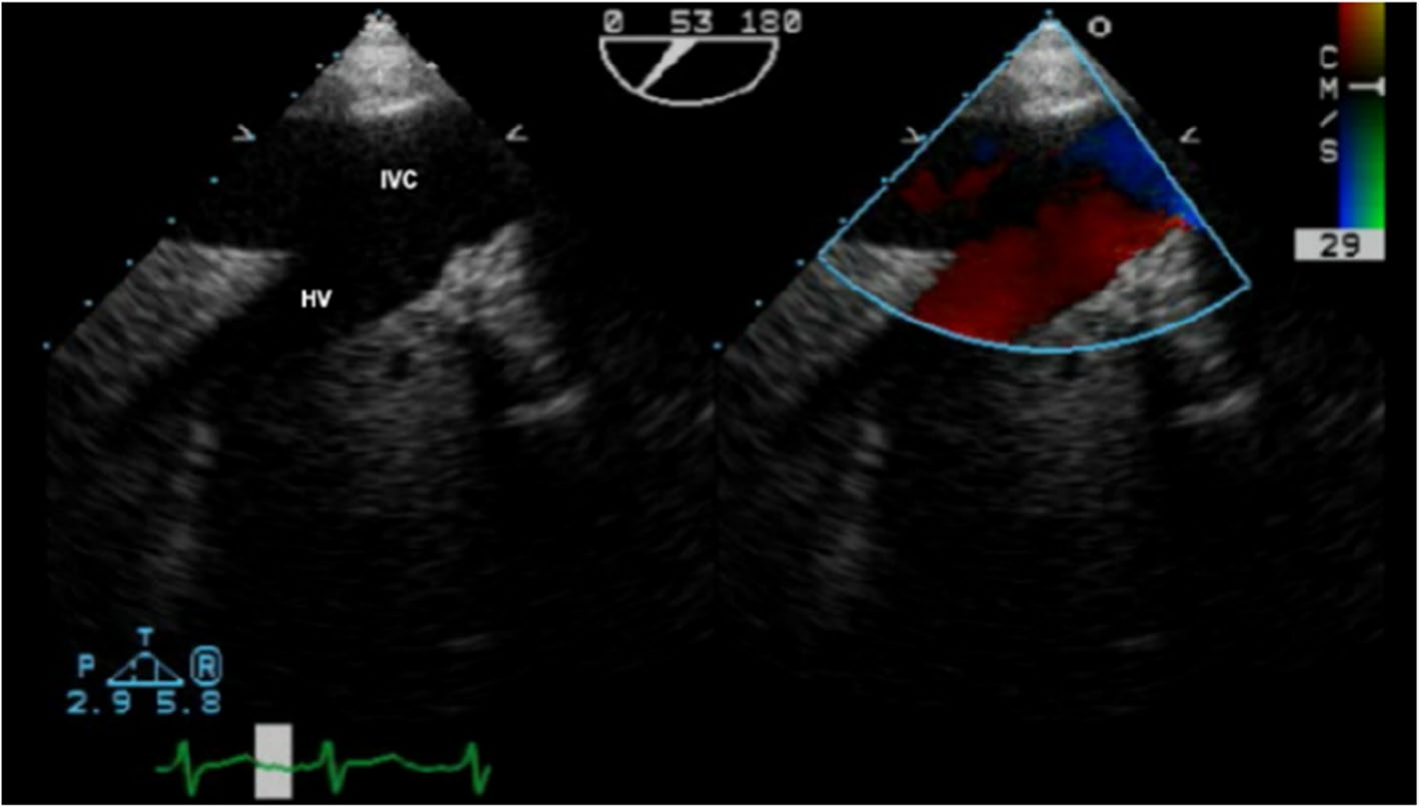
Hepatic vein flow measured with TEE

Cirrhosis with portal hypertension causes widespread vasodilation. Vasodilating substances accumulate in the systemic circulation due to impaired liver function, leading to venous dilation [1, 2] (Fig. 2).

**Fig. 3:**
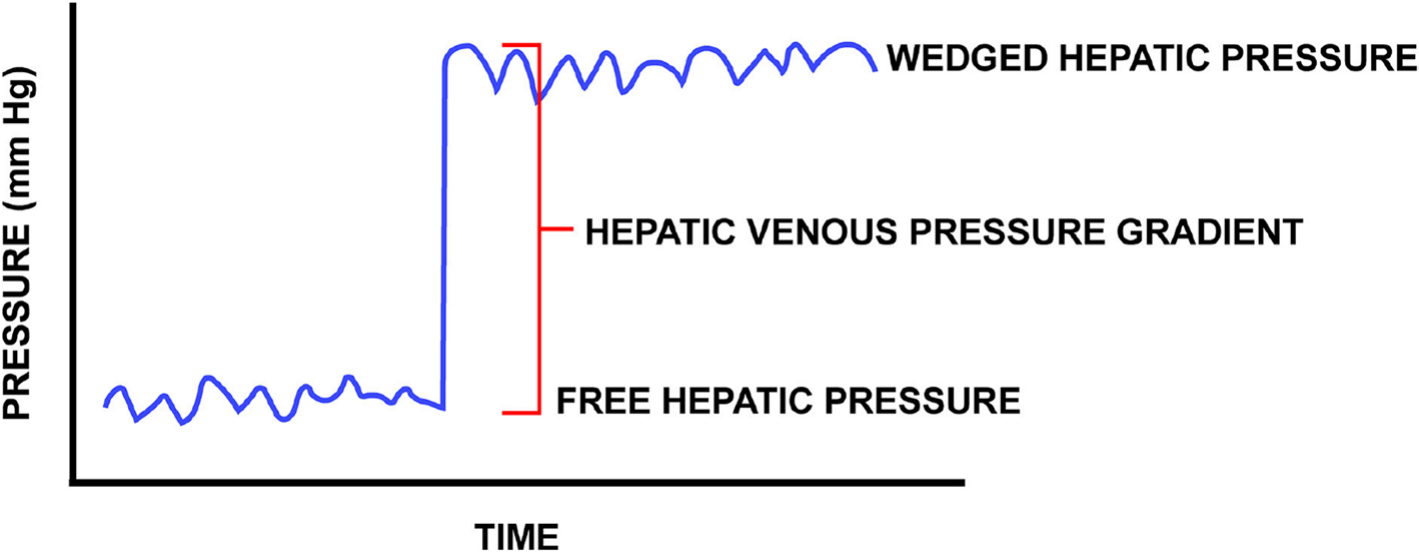
Method for measuring intrahepatic gradient

This results from increased levels of estrogen, prostacyclin, nitric oxide, bradykinin, and vasoactive intestinal peptides, combined with decreased sensitivity to vasoconstrictors. In patients with ESLD, SVR is reduced in all vascular beds [4, 7, 8]. Portal hypertension arises from elevated intrahepatic resistance. Despite this, the splanchnic circulation experiences paradoxical vasodilation due to increased nitric oxide production [4]. Portal hypertension is diagnosed when the pressure gradient between the portal venous pressure and hepatic venous pressure (HVPG) exceeds 5 mmHg [8]. An HVPG greater than 5 mmHg indicates sinusoidal portal hypertension in compensated cirrhosis (Fig. 3).

An HVPG of 10 mmHg or higher signifies clinically significant portal hypertension, predicting disease progression and symptoms. Unlike healthy individuals, cirrhotic patients experience a “steal” of blood from the splanchnic circulation due to elevated hepatic vascular resistance. This can worsen existing hypovolemia, and volume loading often has a limited impact on cardiac output. During liver transplantation, volume loading can exacerbate surgical bleeding, especially during the dissection phase [8].

In patients with ESLD, the hyperdynamic state is characterized by higher cardiac output, heart rate, and blood volume, which initially compensates the drop in peripheral vascular resistance. However, with the progression of liver cirrhosis, arterial compliance increases, SVR decreases, and compensatory mechanisms come to be insufficient. However, as the disease progresses, arterial compliance increases, SVR decreases, and compensatory mechanisms become insufficient [2–4, 7, 9]. As liver failure worsens, the sympathetic nervous system and the renin–angiotensin–aldosterone system (RAAS) are maximally stimulated, but their efforts to increase CO and vasoconstriction are inadequate to maintain effective circulatory volume and counteract splanchnic vasodilation [7]. The splanchnic blood volume becomes disproportionately large compared to the heart, lungs, and aorta, leading to effective hypovolemia [4]. The heart muscle’s structure and function are also compromised.

## Cirrhotic cardiomyopathy

In cirrhotic patients, we observe left ventricular hypertrophy, particularly in the interventricular septum, left ventricular enlargement with an increase in left ventricular end-systolic, and end-diastolic diameters, and additionally, left atrial volume is increased. However, the extent of these changes is typically mild [2, 3].

CCM is characterized by a triad of systolic and diastolic dysfunction and electrophysiological abnormalities. These changes often manifest under stress, not at rest, and occur without underlying heart disease [4, 5, 7]. Accurate data on CCM prevalence is limited due to its asymptomatic nature until the late stages. Cirrhosis often initially presents with subtle left ventricular diastolic dysfunction while maintaining normal systolic function. However, stressors like liver transplantation can reveal cardiac dysfunction. Studies suggest that up to 50% of liver transplant recipients experience cardiac problems, and 7–21% die from heart failure complications post-transplant. Many patients with advanced cirrhosis (e.g., Child–Pugh class B and C) have either diastolic dysfunction or ECG abnormalities [1].

**Fig. 4:**
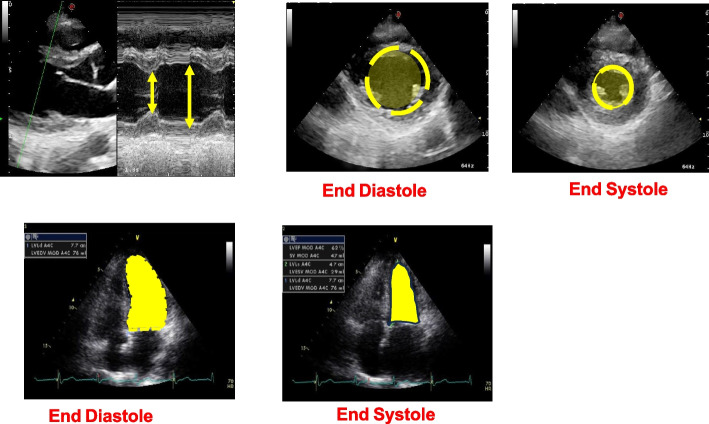
Different methods to evaluate left ventricular function

**Fig. 5:**
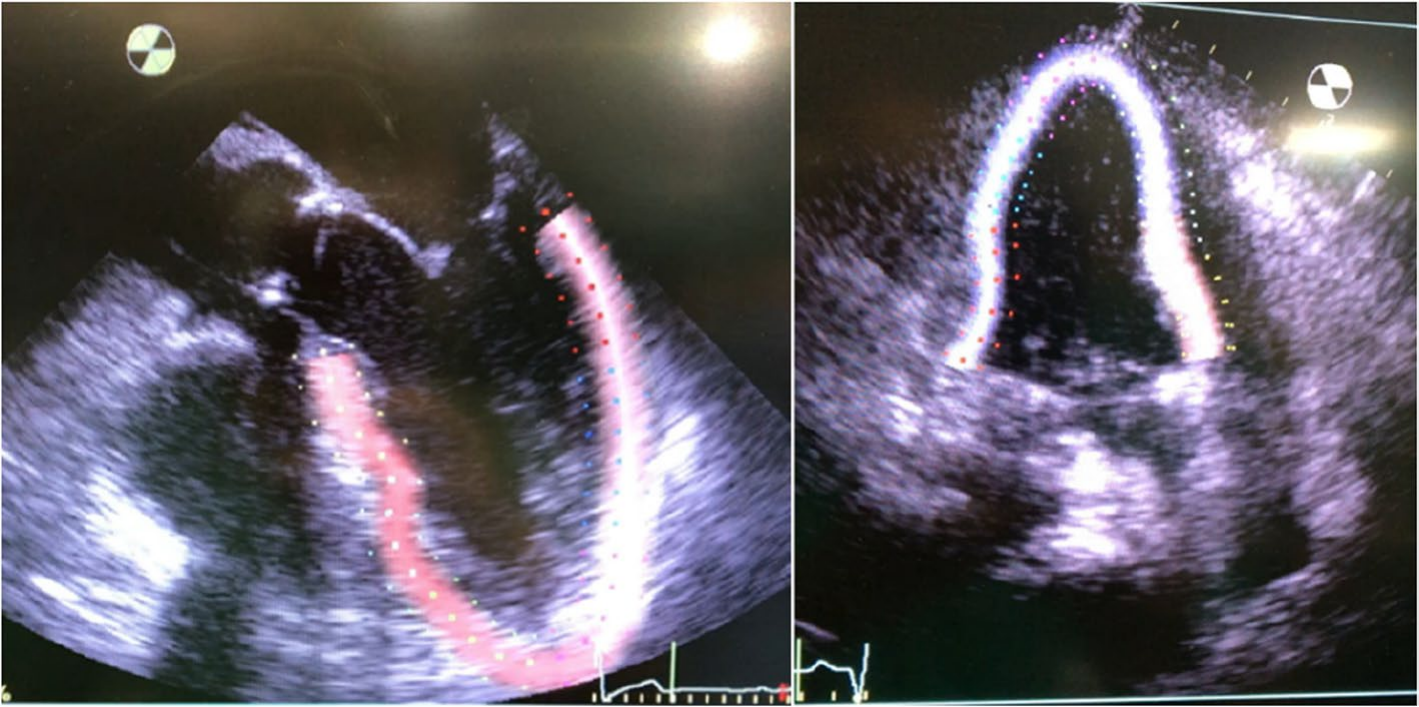
TEE and TTE speckle tracking

To detect these silent lesions, accurate diagnostic tools like two-dimensional echocardiography (Figs. 4 and 5) with tissue Doppler imaging (TDI) and strain imaging are essential, especially for high-risk liver transplant candidates. New diagnostic criteria for CCM were introduced in 2019 [10].

Serial echocardiographic examinations, as reported in Table 1, at 6-month intervals in liver transplant waitlist candidates using TDI and strain imaging are recommended [10].

**Table 1:** CCC Criteria for cardiac dysfunction

Type of dysfunction	2019 CCC criteria
**Systolic dysfunction**	Any of the following is met: - LV ejection fraction < 50% - Absolute GLS < 18% or > 22%
**Diastolic dysfunction**	≥ 3 of the following are met^a^ - Septal e′ velocity < 7 cm/s - E/e′ ratio ≥ 15 - LAVI > 34 ml/m^2^ - TR velocity > 2.8 m/s^2b^
**Supportive criteria or area for future research**	Abnormal chronotropic/inotropic response Electrocardiographic changes Electromechanical uncoupling Serum biomarkers Chamber enlargement CMRI

*CCC* Cirrhotic Cardiomyopathy Consortium, *LV* left ventricle, *GLC* global longitudinal strain, *E* early transmitral flow velocity, *A* late transmitral flow velocity, *e′* early diastolic mitral annular velocity, *LAVI* left atrial volume index, *TR* tricuspid regurgitation, *CMRI* cardiac magnetic resonance imaging

^a^Diagnosed with advanced diastolic dysfunction (grade II or III)

^b^Primary pulmonary hypertension or portopulmonary hypertension should be ruled out

## Two-dimensional speckle tracking echocardiography

Two-dimensional speckle tracking echocardiography (2D-STE) is a promising new imaging technique. 2D-STE utilizes natural acoustic markers, known as “speckles,” visible in conventional grayscale ultrasound images (see Fig. 5). These speckles are unique patterns that arise from the interaction of ultrasound energy with the tissue.

The software tracks these speckles throughout the cardiac cycle and measures the distance between them in multiple regions of interest. This information is used to calculate myocardial deformation and derive myocardial strain, which represents the degree of myocardial fiber shortening. Strain can be expressed in various forms, including global longitudinal strain (GLS), global circumferential strain, and radial strain. However, GLS is the most commonly used indicator of left ventricular (LV) function.

**Fig. 6:**
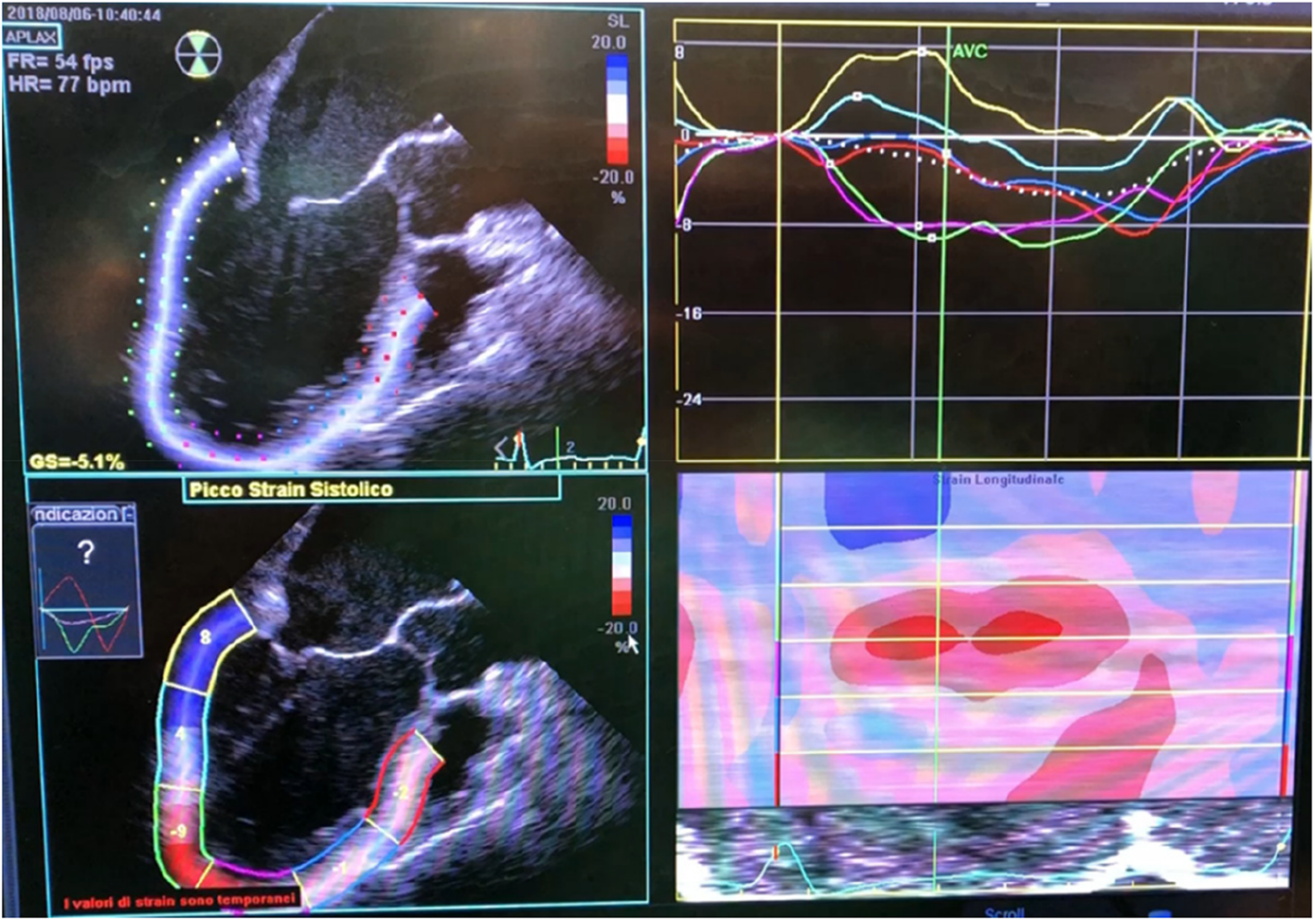
Speckle tracking in cardiac dysfunction

GLS, expressed as a percentage (change in length during systole relative to baseline length at diastole), can identify myocardial dysfunction even in patients with preserved left ventricular ejection fraction (LVEF). An abnormal GLS, defined as greater than − 18% or less than − 22% without cardiac risk factors, is diagnostic for CCM [7, 10] (Fig. 6).

Additionally, if an ESLD patient has a known reduced ejection fraction (< 50%) without cardiac risk factors, this is also indicative of CCM. 2D-STE can distinguish between active and passive myocardial tissue movement, such as those in areas with scars or infarctions. It can also evaluate cardiac rotation, twisting, and torsion. The application of 2D-STE has enabled a more detailed characterization of the echocardiographic changes observed in CCM [7, 10]. Recent evidence supports the use of 2D-STE for pre-operative cardiac assessment in liver transplant patients. Hyperdynamic left ventricular contractility, particularly in Child–Pugh class C patients, was found to be an independent predictor of reduced transplant-free survival. Another study revealed that abnormally low or high GLS values were strongly predictive of post-operative mortality following LT. “Low” GLS was defined as less negative than − 18% in females, than − 17% in males. “High” GLS was defined as more negative than − 26% in females, than − 24% in males. While a low GLS (indicating low contractility) intuitively correlates with complications, surprisingly, those complications are also observed with a high GLS (indicating high contractility). This suggests that a highly contractile state may be present in the earlier, pre-decompensated stages of cirrhotic cardiomyopathy. 2D-STE has some disadvantages, including the need for relatively expensive software and variations in measurements obtained using different vendor software [7, 10]. 

## Diastolic dysfunction

The hearts of patients with cirrhosis often undergo morphological changes, including cardiac hypertrophy and patchy areas of fibrosis and subendothelial edema. These alterations can contribute to both systolic and diastolic dysfunction [1–3]. In patients with ESLD, diastolic dysfunction serves as a warning sign for CCM. Notably, diastolic dysfunction typically precedes systolic dysfunction [4].

Diastolic dysfunction is prevalent in patients with liver cirrhosis, with reported rates ranging from 25.7 to 81.4%. It is associated with increased mortality and adverse outcomes, including higher allograft rejection, graft failure, and heart failure post-liver transplantation [2, 4].

Sodium retention and an overactive RAAS may contribute to diastolic dysfunction in patients with cirrhosis [1–3]. Abnormal left ventricular relaxation impedes ventricular blood flow, leading to increased left ventricular end-diastolic pressure and reliance on atrial contribution for late ventricular filling. Echocardiographic Doppler parameters can easily assess diastolic function.

**Fig. 7:**
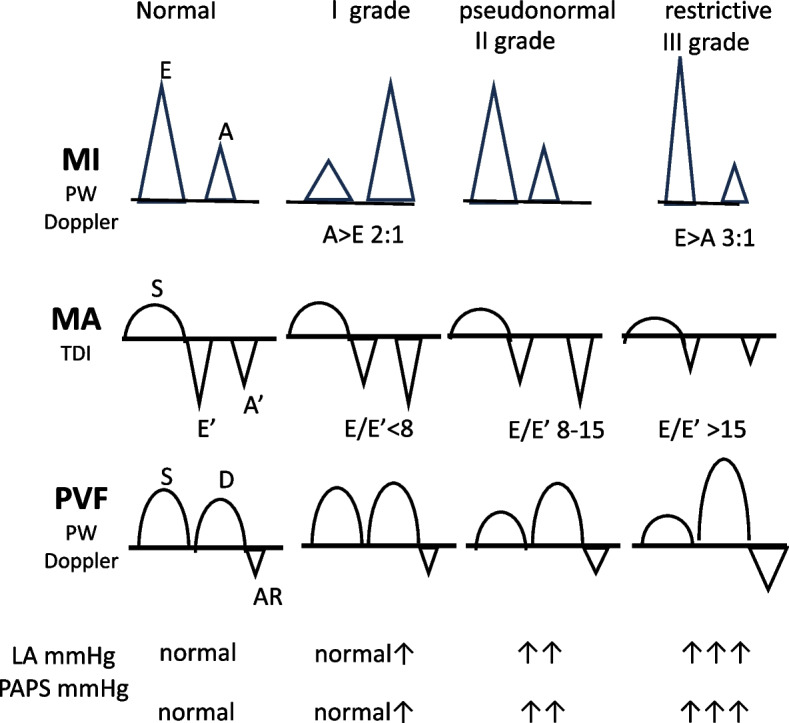
Methods to evaluate diastolic function

Decreased left ventricular compliance and relaxation result in an abnormal filling pattern, characterized by delayed trans-mitral blood flow despite increased atrial contribution. These hemodynamic changes are reflected in the echocardiogram as a decreased E/A ratio or increased E/e′ ratio [3]. The E wave represents early passive trans-mitral flow, while the A wave represents trans-mitral flow driven by atrial contraction [1, 4] (Fig. 7).

Cardiac preload significantly impacts the development of diastolic dysfunction. Variations in preload influence the progression of diastolic compliance impairment. Patients with ascites who undergo large-volume paracentesis often experience substantial improvement in diastolic dysfunction due to increased blood return to the heart. The E/A ratio is a commonly used marker for assessing diastolic dysfunction, but it has limitations in sensitivity and specificity due to its dependence on preload and afterload.

Diastolic dysfunction is not directly linked to the etiology of liver disease and is not dependent on the stage of liver disease. However, its severity correlates with the degree of liver failure, suggesting a relationship between diastolic dysfunction grade and Child–Pugh score [2, 3]. Diastolic dysfunction in patients with ascites can be significant and may improve after paracentesis [3]. Importantly, diastolic dysfunction affects the prognosis of patients undergoing transjugular intrahepatic portosystemic shunt (TIPS) insertion or liver transplantation. The cirrhotic heart may struggle to adapt to increased preload after TIPS insertion due to diastolic dysfunction, leading to an enlarged left atrial diameter and elevated pulmonary capillary wedge pressure. However, diastolic volumes may normalize over time, although mild left ventricular hypertrophy may persist [1, 3].

**Fig. 8:**
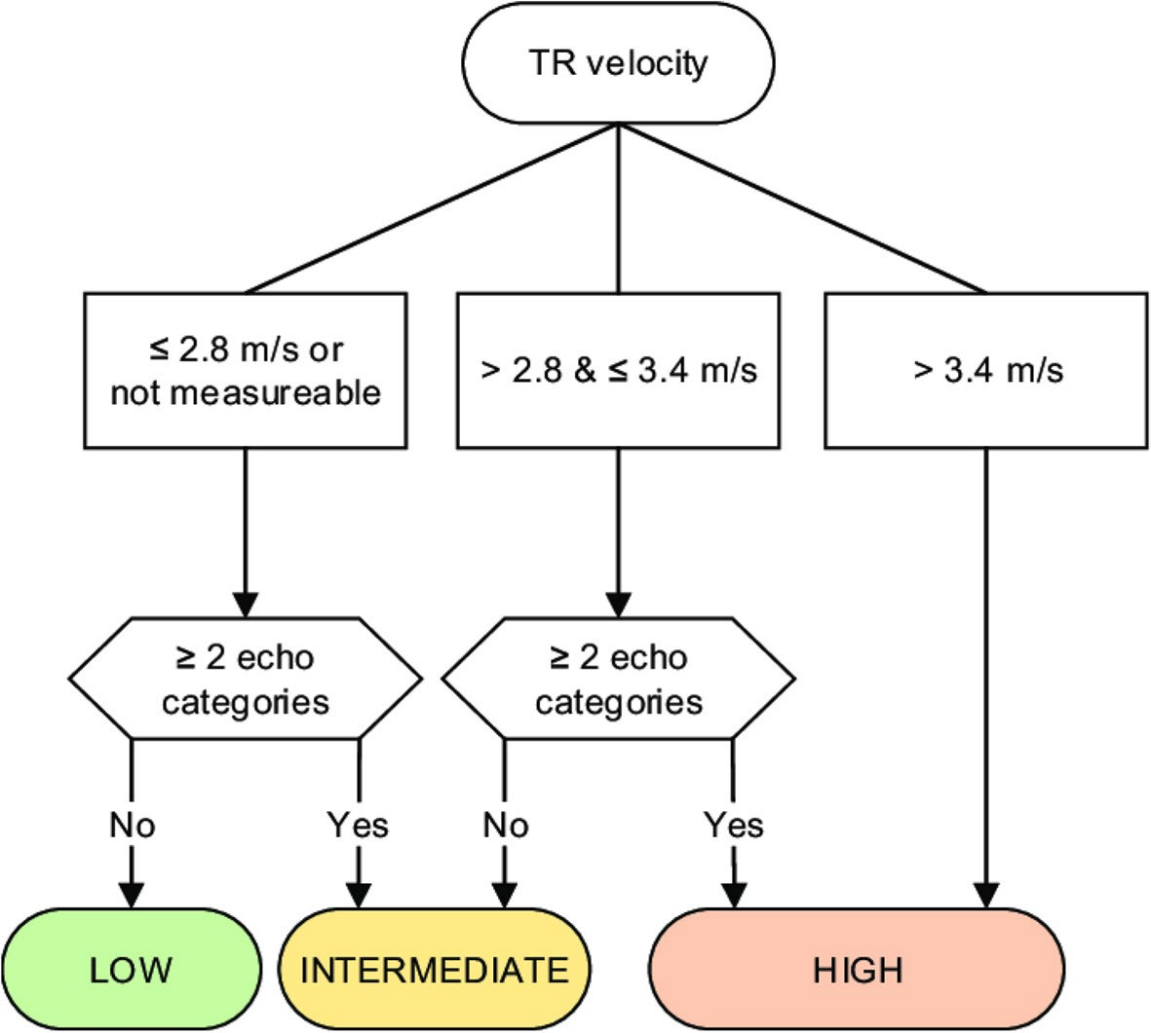
Algorithm for diagnosis of pulmonary hypertension from TR Doppler velocity

The 2016 American Society of Echocardiography and European Association of Cardiovascular Imaging (ASE/EACVI) guidelines for diastolic dysfunction exploit the following parameters, which are also adopted in the updated diagnostic criteria for CCM by the Cirrhotic Cardiomyopathy Consortium (CCC): septal < 7 cm/s or lateral < 10 cm/s early diastolic mitral annular velocity (e′), E/e′ ratio > 14, left atrial volume index > 34 mL/m^2^ and tricuspid regurgitation > 2.8 m/s velocity [2, 6] (Fig. 8).

The parameters used in the 2016 ASE/EACVI guidelines for diastolic dysfunction, adopted by the CCC, are superior to previously used metrics such as E/A ratio, deceleration time, and isovolumetric relaxation time. These newer parameters can independently indicate the presence of diastolic dysfunction, while the older metrics are only useful when concordant.

Assessing diastolic dysfunction is challenging in patients with mitral valve disease, mechanical valves, and atrial fibrillation. However, it remains a valuable tool with broad applicability in most patients [7].

## Systolic dysfunction

Systolic dysfunction is a component of the diagnostic criteria for CCM, but it is often masked by the hyperkinetic circulation in cirrhotic patients, making it difficult to detect. While systolic dysfunction may be present, the hyperdynamic state can obscure it until stress occurs. The decline in systolic function involves the downregulation of β-receptors and the presence of cardio-depressant substances, such as tumor necrosis factor-α, bile acids, endotoxins, cytokines, carbon monoxide, and endogenous cannabinoids [1, 3, 4].

The LVEF is a commonly used parameter for assessing systolic function, but it has limitations. LVEF can be significantly influenced by loading conditions, potentially masking systolic dysfunction when afterload is low, as in cirrhotic patients. GLS measures the percentage of systolic myocardial shortening in the longitudinal direction. GLS has been shown to be more effective in detecting subclinical systolic dysfunction, especially when LVEF is within the normal range [2].

According to the definition of CCM, systolic dysfunction is characterized by a blunted increase in cardiac output in response to exercise, volume challenges, pharmacological stimuli, or a resting LVEF < 55%. In CCM patients, heart failure may only manifest under hemodynamic stress. The LVEF response to exercise is impaired in patients with cirrhosis. During exercise, left ventricular end-diastolic pressure increases, but the expected increases in cardiac stroke index and LVEF are blunted, indicating an inadequate ventricular reserve response to increased filling pressure.

Similar responses can be observed after TIPS insertion, with approximately 12% of patients developing heart failure. However, cardiac pressures typically normalize over time. Pharmacological cardiac strain using vasoconstrictors like noradrenaline, vasopressin, glypressin, and inotropes like dobutamine can also lead to systolic dysfunction, with increases in left ventricular end-diastolic volume and pressure and reduced LVEF [3].

Six to twelve months after liver transplantation, significant improvements in cardiac performance, response to physical stress, and normalization of cardiac output and myocardial mass are often observed.

In conclusion, systolic dysfunction with reduced LVEF can occur in the context of hyperdynamic circulation. It may be diagnosed at rest or during exercise. In the late stages of liver disease, declining systolic function is associated with complications and survival. Systolic function changes significantly after LT [3].

## Portopulmonary hypertension

PoPH is a serious complication of portal hypertension, affecting 1.1–6.3% of patients with this condition. It constitutes 5–16% of all pulmonary arterial hypertension (PAH) cases [7, 11]. PoPH is diagnosed when pulmonary arterial hypertension is present in individuals with confirmed cirrhosis-related portal hypertension [2, 7]. Non-cirrhotic causes of portal hypertension leading to PoPH include portal vein thrombosis, granulomatous disease, autoimmune diseases, drug reactions, infections like hepatitis C, and congenital abnormalities such as congenital portosystemic shunts. In LT patients, the prevalence of PoPH ranges 5–10% [11, 12]. PoPH is characterized by a mean pulmonary arterial pressure (mPAP) > 25 mmHg at rest, pulmonary arterial wedge pressure < 15 mmHg, pulmonary vascular resistance (PVR) > 3 Wood Units, or 240 dynes/s/cm^−5^ associated with cirrhotic or non-cirrhotic portal hypertension [2, 5, 7, 12, 13]. The severity of cirrhosis, as measured by the Child–Pugh Score or Model of End-Stage Liver Disease (MELD score), was not correlated with the presence or severity of PoPH [2, 12]. Patients with cirrhosis and PoPH have a very poor prognosis. Without liver transplantation or PAH-specific therapies, only 14% of PoPH patients survive for 5 years. Data from the Multicenter Liver Transplant Database indicates a mortality rate of 36% among patients with PoPH undergoing liver transplantation [7, 13].

The exact mechanisms underlying the development of PoPH are not fully understood. However, it is believed to arise from vascular constriction or obstruction of the pulmonary arterial bed, resulting in increased PVR [7]. A case series examining autopsy results identified several contributing factors to PoPH, including portal hypertension, pulmonary artery thromboembolism, endothelial cell proliferation, and platelet aggregation. The pathological changes in the pulmonary vasculature observed in PoPH are similar to those seen in other forms of idiopathic PAH. These changes include intimal fibrosis, increased medial thickness, in situ thrombosis, plexiform lesions, and hyper-proliferation of smooth cells, which obstruct the pulmonary arterioles.

PoPH differs from other forms of PAH in its presentation of preserved or even high CO with increased PVR. Portal hypertension represents a state of hyperdynamic circulation due to splanchnic vasodilation and the formation of portosystemic shunts [9].

PoPH is primarily driven by two mechanisms:

1. Hyperdynamic circulation in cirrhosis: The increased pulmonary blood flow associated with hyperdynamic circulation exposes the pulmonary vasculature to elevated shear stress, leading to endothelial cell injury and remodeling of the pulmonary vascular bed [5, 9, 12]. This results in the progressive obliteration of small pulmonary arteries, increasing PVR and straining the right ventricle, potentially leading to right ventricular failure [12].
2. Vasoactive substance shunting: The shunting of vasoactive substances from the splanchnic circulation to the pulmonary circulation causes progressive pulmonary vascular vasoconstriction and remodeling. Various metabolites, such as endothelin-1, serotonin, and glucagon, directly or indirectly injure the pulmonary endothelium and arterial smooth muscle [2, 5, 9, 12]. Among these, endothelin plays a particularly crucial role in promoting pulmonary vasoconstriction and remodeling [9].

Only a minority of patients with portal hypertension develop PoPH. Genetic, immune, and environmental factors likely contribute to the changes in pulmonary circulation. Further research is needed to identify specific predictors. Female sex and autoimmune hepatitis are risk factors, while ESLD secondary to hepatitis C is associated with a decreased risk [5, 9].

Hemodynamic changes induced by portal hypertension drive pulmonary remodeling in PoPH [9]. Diagnosis of PoPH requires a high clinical suspicion. Patients with PoPH may remain asymptomatic for months to years, and symptoms are often similar to those of other associated conditions. Dyspnea, weakness, fatigue, edema, orthopnoea, palpitations, and chest pain with exertion are common. Syncope may occur in severe cases.

Physical examination may reveal jugular venous distension, a loud S2 heart sound, a left parasternal systolic murmur, peripheral edema, and ascites. Chest radiography findings are often non-diagnostic. Electrocardiographic findings may include right bundle branch block, right axis deviation, right ventricular hypertrophy, and precordial T-wave inversions. Mild to moderate hypoxemia and hypocapnia are common, but overt signs of cyanosis and clubbing are usually absent. Pulmonary function tests do not follow a specific ventilatory pattern but often show reduced lung volumes, especially diffusion capacity [5, 9, 13].

**Fig. 9:**
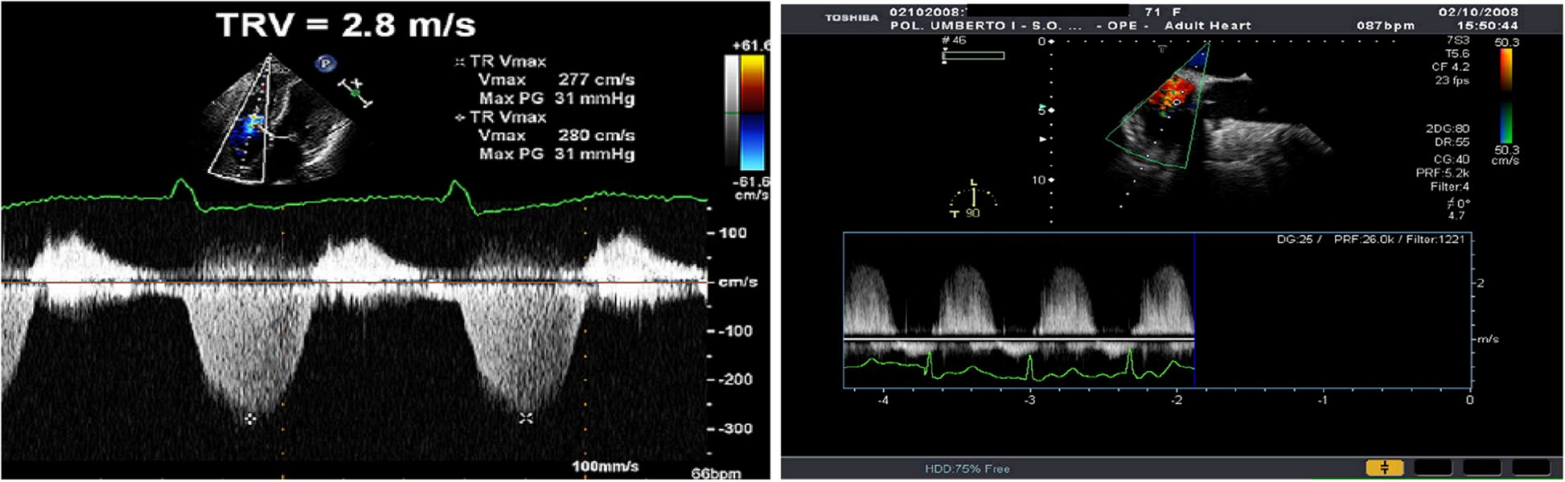
TTE and TEE measure of PAPS

**Fig. 10:**
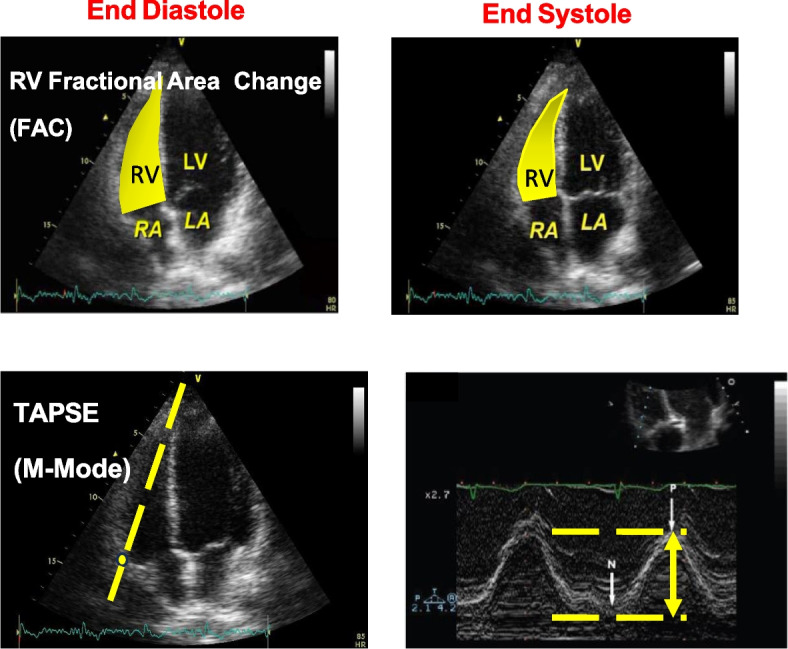
Methods to evaluate right ventricular function

Echocardiography is the preferred screening tool for PoPH. All liver transplant candidates and symptomatic patients with liver disease or portal hypertension should undergo Doppler echocardiography to screen for elevated pulmonary pressures, as PoPH has prognostic significance. Using the peak tricuspid regurgitant velocity (TRV), estimated Right Atrial pressure (RAP), and the modified Bernoulli equation, right ventricular systolic pressure (RVSP) can be calculated as (Fig. 9) and accompanied by an assessment of right ventricular function (Fig. 10):

$${4\left(\mathrm{TRV}\right)}^2\;+\;\mathrm{RAP}$$

In the absence of pulmonic stenosis, pulmonary systolic pressure can be estimated using the RVSP. Patients with a systolic Pulmonary artery pressure (PAP) value of 30 mmHg or higher are typically diagnosed with PoPH [2, 5, 9]. Transthoracic echocardiography (TTE) provides valuable information about indirect signs of PAH, such as right ventricular dilatation, hypertrophy, and abnormal septal motion [13].

TTE has shown high sensitivity and specificity (97% and 77%, respectively) in screening LT candidates for PoPH. Approximately 20% of LT candidates have moderately elevated pulmonary pressure, but only 5–10% have PoPH. The remaining cases may be due to hyperdynamic circulation, volume overload, or LV dysfunction. This distinction is crucial, as PoPH patients have increased risks during LT.

Right heart catheterization (RHC) is recommended to confirm and stage PoPH when an RVSP of 45–60 mmHg or evidence of right ventricular hypertrophy or dysfunction is found on TTE [5, 9, 14].

**Fig. 11:**
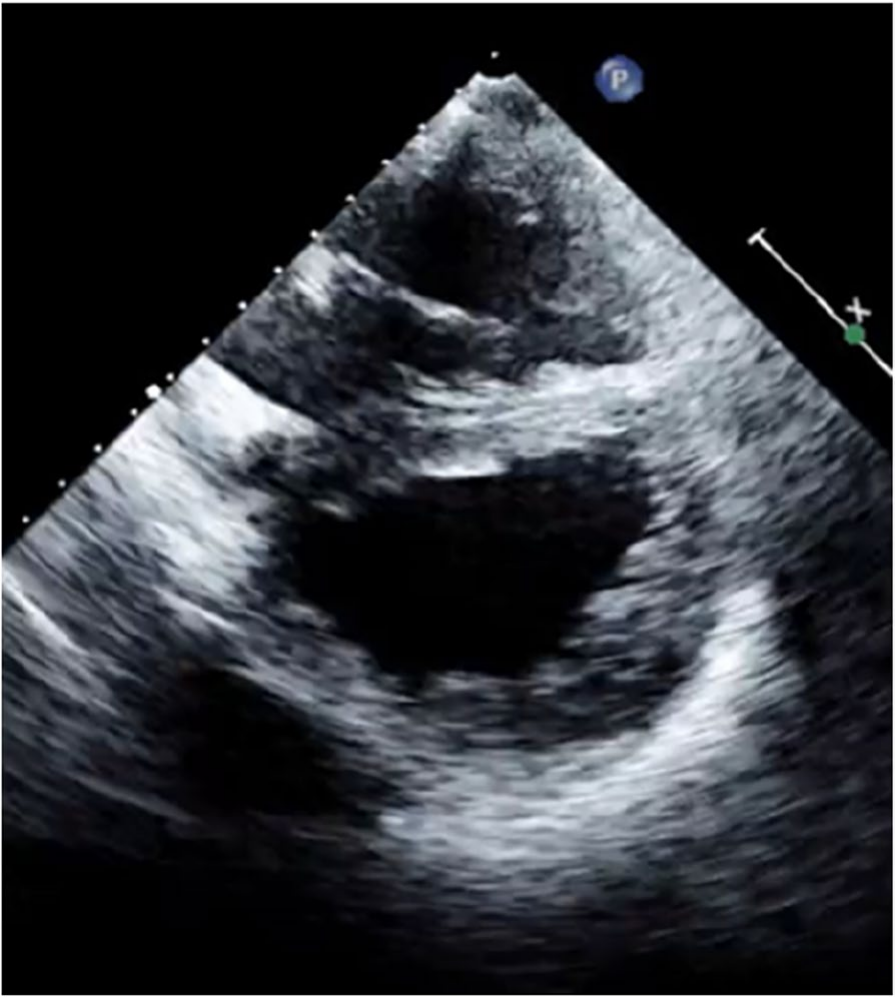
Severe pulmonary hypertension with D shape left ventricle

The mPAP, measured either indirectly with echocardiography or directly with RHC, also indicates the severity of PoPH. PoPH is classified as mild (35 mmHg), moderate (35–45 mmHg), or severe (45 mmHg) [9] (Fig. 11).

The severity of PoPH is crucial, as a mPAP of 35 mmHg or higher carries an increased risk of peri- and post-operative mortality in liver transplant candidates. This threshold is also considered the benchmark for initiating PoPH-specific therapy (Fig. 11). RHC provides valuable data to exclude other causes of PAH in liver transplant candidates.

By assessing PVR, CO, mean PAP, and other parameters, the nature and severity of PoPH can be characterized. For example, in cases of left heart disease, CO is reduced, while PVR and PCWP are increased. In contrast, patients with PoPH often have low CO and PCWP with elevated PVR. In the hyperdynamic state, CO increases, PCWP is normal, and PVR decreases. The two most commonly used methods for determining CO are the thermodilution and Fick methods, which demonstrate a reasonable correlation at high CO states. The trans-pulmonary gradient, calculated as the difference between PCWP and mPAP, can help identify pulmonary venous hypertension. A gradient exceeding 12 mmHg suggests pulmonary venous hypertension, which does not appear to increase mortality risk in liver transplantation [9, 13].

The treatment and prognosis of these conditions vary significantly, emphasizing the importance of accurate diagnosis. Patients with an elevated mean PAP but normal PVR (hyperdynamic state) can benefit from liver transplantation without further catheterization or treatment. The trans-pulmonary gradient is calculated as the mean PAP-PCWP [13]. Recent studies have shown that pharmacotherapy can reduce pulmonary pressures in some patients with cirrhosis, facilitating successful liver transplantation [5].

A significant proportion of patients with cirrhosis and an RVSP of 50 mmHg on echocardiography may have normal PVR values on RHC. This difference can be attributed to cirrhotic-related volume overload or hyperdynamic circulation.

Advances in medical therapy and liver transplant surgery have significantly improved survival outcomes for patients with PoPH and advanced liver disease. Given the prognostic importance of PoPH and the limited donor pool, a comprehensive pre-operative cardiopulmonary assessment is crucial in patients with pre-transplant cirrhosis. Patients with mild PoPH can safely undergo liver transplantation, while those with moderate-to-severe PoPH should undergo RHC and consider vasodilator therapy [2, 13] (Table 2).

**Table 2:** Grading for portopulmonary hypertension

Summary on assessment, grading, and therapy for PoPH	
**TTE in all patients prior to transplant surgery, systolic PAP > 30 mmHg** **RHC in selected cases, confirmation and grading of PoPH**	
**Mild PoPH**	mPAP > 25 mmHg, PVR > 240 dyn-s-cm^5^, PAWP < 15 mmHg
**Moderate PoPH**	mPAP 35–45 mmHg, PVR > 240 dyn-s-cm^5^, PAWP < 15 mmHg if responder to vasodilator therapy, allow LT
**Severe PoPH**	mPAP > 45 mmHg, PVR > 240 dyn-s-cm^5^, PAWP < 15 mmHg, medical therapy

LT should be considered in cases with a positive response to therapy. Bridging therapy with PAH medications should be used until transplantation and continued as needed during the peri- and post-operative periods [2, 13].

## Hepatopulmonary syndrome

HPS is a pulmonary vascular disorder characterized by deep hypoxia due to intra-pulmonary vascular dilation (IPVD) in the context of liver dysfunction [5, 7]. It affects 15–30% of liver transplant candidates and is associated with higher mortality and morbidity in cirrhotic patients. While HPS itself is not a direct cause of death, the progression of cirrhosis and associated complications contributes to adverse outcomes. LT is the only effective treatment for HPS, and it can manifest as intra-operative and post-operative hypoxia, often requiring prolonged mechanical ventilation [2].

The etiology of HPS is believed to stem from cirrhosis-related damage to the pulmonary vascular endothelium, leading to pre- and post-capillary dilation and subsequent shunting. Unlike PoPH, which is caused by pulmonary vasoconstriction, HPS results from pulmonary vasodilation in the setting of liver disease (Table 3).

**Table 3:** Definition and staging of HPS

**Hepatopulmonary syndrome definition**	
** Oxygenation**	PaO_2_ < 80 mmHg or A-a gradient > 15 mmHg or 20 mmHg if age > 64 years while breathing room air
** Intrapulmonary vasodilation**	Confirmed by contrast-enhance echocardiography
** Liver disease**	Cirrhosis and/or portal hypertension
**Staging based on severity of hepatopulmonary syndrome**	
*** Stage***	***Partial pressure of oxygen (mmHg) on room air***
** Mild**	≥ 80
** Moderate**	≥ 60 to < 80
** Severe**	≥ 50 to < 60
** Very severe**	< 50 on room air or > 300 while breathing 100% oxygen

**Fig. 12:**
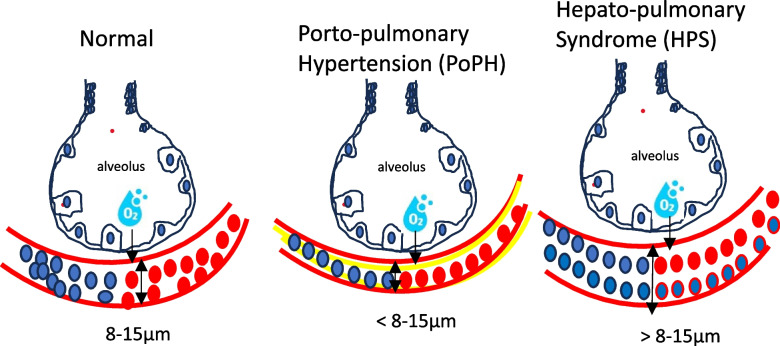
Different morphology of capillary pulmonary vessels in HPS

Though HPS and PoPH share similar clinical presentations, their underlying mechanisms differ significantly. The hypoxemia in HPS is primarily due to diffusion defects caused by the dilated pulmonary vessels, leading to limited oxygen diffusion and disequilibrium. This results in mixed venous blood passing through the pulmonary veins due to increased blood flow through the IPVD [15] (Fig. 12).

Excessive IPVD in HPS leads to ventilation/perfusion mismatch, inadequate oxygenation, intra-pulmonary shunting, cyanosis, and arterial hypoxia [5].

HPS is characterized by the dilation of pulmonary pre-capillary and capillary vessels, along with an increase in the number of these dilated vessels. Other findings include enlarged paraumbilical vein and hepatic artery, lung and pleural spider nevi, and intrahepatic vascular changes such as thrombosis, fibrous septa, and centrilobular venous thickening. Doppler ultrasonography reveals hepato-jugular flow and reduced portal blood flow.

The underlying cause of HPS is believed to be a loss of pulmonary capillary vessel tone and inhibition of pulmonary vasoconstrictors, with nitric oxide playing a significant role in pulmonary vasodilation. Methylene blue, an NOS inhibitor, can transiently improve oxygenation [15].

**Fig. 13:**
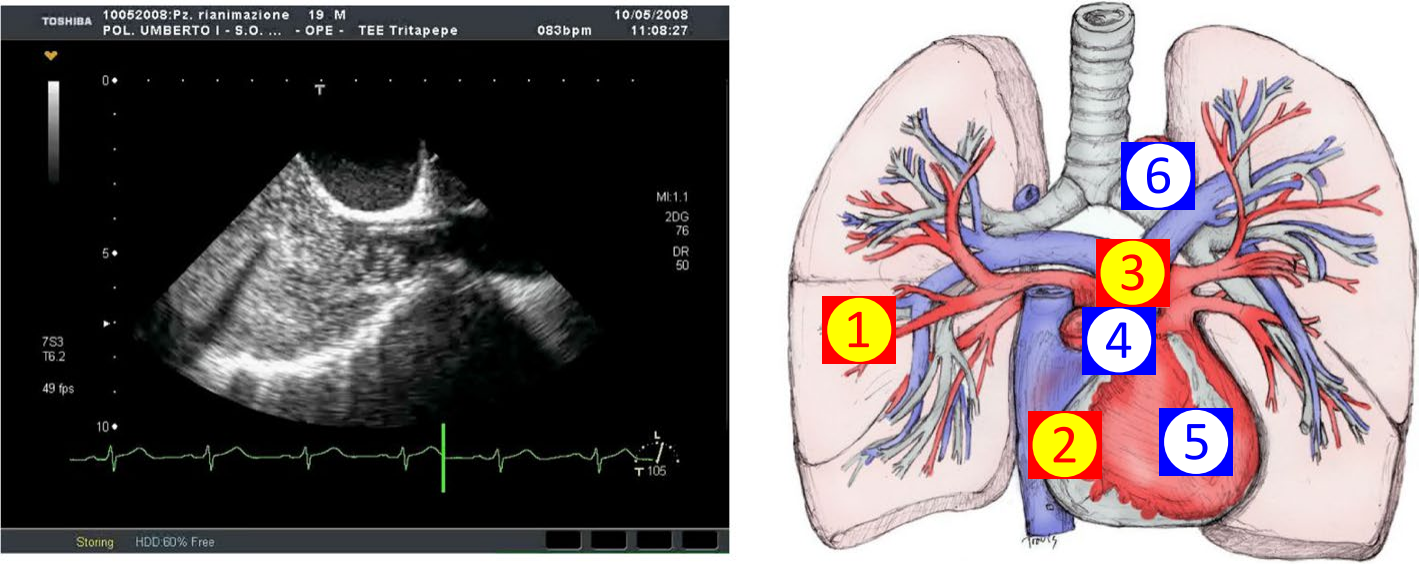
Aschematic view of agitated saline injection test. Bubbles should not pass through the pulmonary vasculature and should be trapped in healthy subjects (1, 2, 3), but in patients with shunts such as patients with hepatopulmonary syndrome, they will appear in the left heart and the systemic circulation subsequently (4, 5, 6)

HPS is diagnosed based on evidence of chronic liver disease, resting hypoxemia, and intra-pulmonary shunting. In the absence of other cardiopulmonary diseases, an alveolar-arterial gradient of 15 mmHg or more (20 mmHg in patients over 65) or an arterial partial pressure of oxygen below 80 mmHg on ambient air, combined with evidence of intra-pulmonary shunting on echocardiography, is diagnostic. Transthoracic contrast echocardiography (TTCE) is the gold standard for demonstrating an intra-pulmonary shunt (Fig. 13).

TTCE is performed by injecting agitated saline into the venous system during echocardiography. The agitated saline creates microbubbles that are visualized in the right atrium and then filtered by the pulmonary capillary bed. Normally, these microbubbles do not pass through the pulmonary capillaries. However, in the presence of intra-cardiac or intra-pulmonary shunts, microbubbles can be visualized in the left heart chambers. The timing of microbubble appearance in the left atrium varies with heart rate, cardiac output, and shunt size.

With an intra-pulmonary shunt, microbubbles appear in the left atrium within 4–6 beats, or possibly 8–10 beats with depressed cardiac output. With an intra-cardiac shunt, microbubbles appear in the left atrium within the first three beats. The pressure gradient between the right and left atria affects their visualization. Performing contrast-enhanced echocardiography in the standing position can improve results [2, 5, 7, 15].

Technetium-99 m-labeled macroaggregated albumin perfusion scanning can also diagnose HPS. In normal conditions, most of the labeled albumin is trapped in the pulmonary circulation. However, in the presence of intra-pulmonary shunting, some albumin escapes into other organs, such as the brain, liver, and spleen. Unlike TTCE, this method cannot differentiate between intra-pulmonary and intra-cardiac shunts. Atrial septal defects must be excluded, as they can also cause isotope appearance in the liver.

Pulmonary angiography is rarely used to diagnose HPS and is generally indicated only in patients suspected of having pulmonary arterio-venous malformations, which are uncommon in HPS [5, 15]. (Table 4).

**Table 4:** Comparison of the diagnostics of PoPH and HPS

Diagnostic criteria for portopulmonary hypertension	Diagnostic criteria for hepatopulmonary syndrome
Presence of portal hypertension	Presence of portal hypertension, chronic liver disease, or congenital porto-systemic shunts
Presence of pulmonary arterial hypertension (PAH) according to the following hemodynamic criteria from right heart catheterization: - Mean pulmonary arterial pressure (mPAP) > 20 mmHg - Pulmonary artery wedge pressure (PAWP) ≤ 15 mmHg - Pulmonary vascular resistance (PVR) ≥ 240 dyne/s/cm^5^ or 3 Wood Units (WU)	Presence of intrapulmonary vascular dilatation in the contrast-enhanced transoesophageal echocardiography (CE-TTE) (microbubbles in the left heart ≥ 3 beats after right heart microbubbles) Abnormal arterial oxygenation defines by an elevated alveolar-arterial oxygen gradient (AaO_2_) ≥ 15 mmHg (or > 20 mmHg if age > 64 years)

Transesophageal echocardiography is a more specific alternative to TTCE. However, it is often avoided due to the high risk of esophageal variceal bleeding in this patient population [15].

Available medical therapies for HPS have limited effectiveness. The definitive treatment for HPS remains liver transplantation, which has been shown to significantly improve mortality rates in patients with this condition [15].

## Left ventricular outflow tract obstruction, coronary artery disease, and pericardial effusion

Echocardiography can be used to assess left ventricular outflow tract obstruction (LVOTO), valvular heart disease, pericardial effusion, and underlying coronary artery disease (CAD) using dobutamine stress echocardiography (DSE). This evaluation is valuable prior to LT. DSE can screen for CAD, simulating the hemodynamic stress of LT. However, its utility as a screening tool in this population is doubtful [5].

Coronary angiography remains the gold standard for CAD diagnosis but carries risks for patients with ESLD, including increased bleeding risk and potential contrast-induced renal failure [5]. It should be considered for LT candidates with known CAD but not used as the initial screening test.

**Fig. 14:**
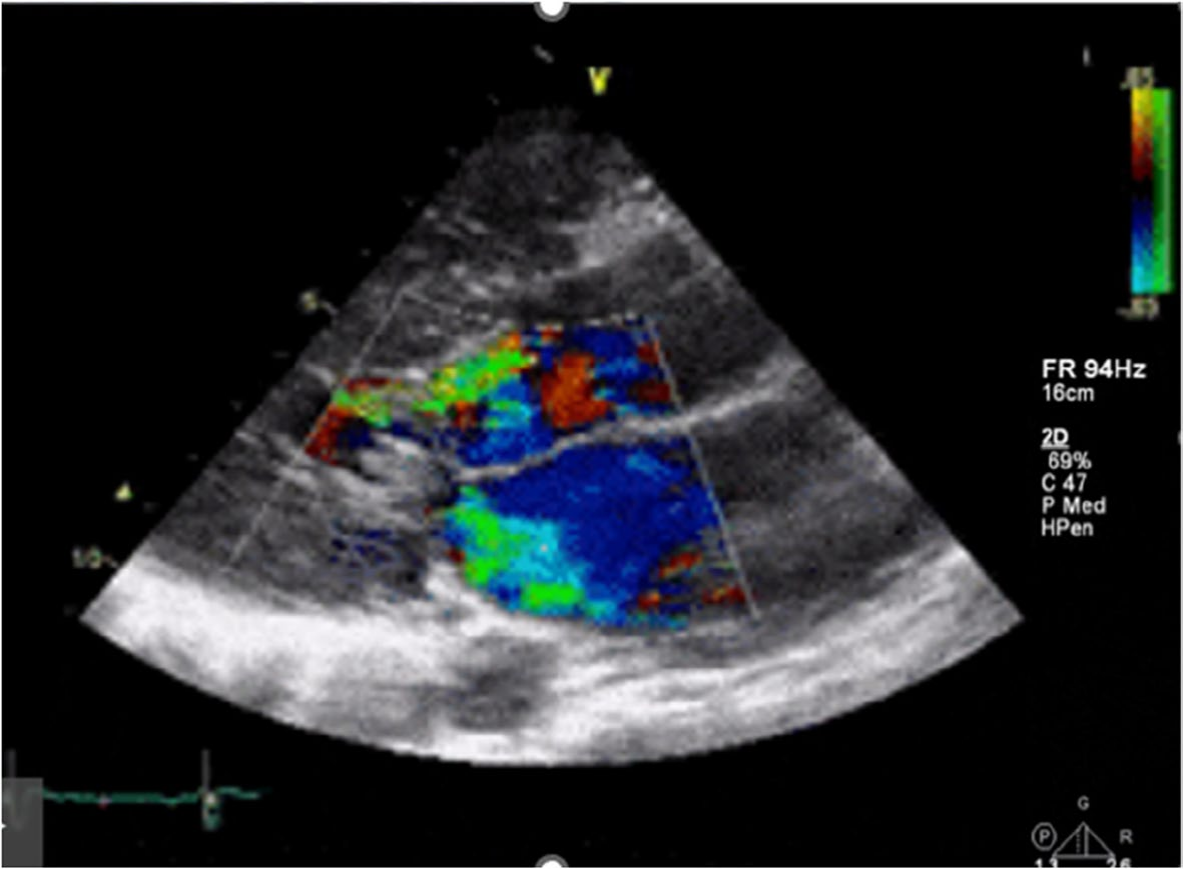
LVOTO with mitral insufficiency and increase of aortic gradient

LVOTO is defined as a peak Doppler pressure gradient of 30 mmHg or more and is considered hemodynamically significant when exceeding 50 mmHg. While typically associated with hypertrophic cardiomyopathy or acute myocardial infarction, LVOTO can also occur in conditions like decreased preload, afterload, increased heart rate, and contractility, which are similar to pathophysiological changes in cirrhotic patients. Intraoperative factors like blood loss, decreased systemic vascular resistance, sympathetic nervous system activation, and inotropic agent use can induce LVOTO. Careful intra-operative monitoring is necessary to avoid hypovolemia, tachycardia, and inotropic agent use [2, 5] (Fig. 14).

LT candidates often have fluid retention, manifesting as peripheral edema, ascites, pleural effusions, and pericardial effusion. Pericardial effusions are reported in up to 63% of patients with ESLD, but they are typically small and well-tolerated. Echocardiography should be used to assess the size of the effusion and the presence of hemodynamic compromise, which can be evaluated by chamber collapse and characteristic alterations in mitral and tricuspid flow. Pre-emptive drainage may be considered if a large effusion with a potential risk of hemodynamic compromise is identified [2].

## Conclusions

Liver cirrhosis has a substantial effect on the cardiovascular system; this results in high portal vein pressure as well as alterations in systemic vascular resistances and blood volume circulation. All these changes have a direct effect on heart function.

Assessing cardiac risk in patients with ESLD, especially those undergoing LT, is crucial. Cardiovascular complications are a major cause of mortality and morbidity associated with LT. CCM, PoPH, and HPS are potential complications of liver disease. Understanding these conditions is essential for effective patient management and improved outcomes, particularly in LT.

Echocardiography is a valuable tool for clinicians managing patients with cirrhosis, a complex disease affecting multiple vital organs.

## Data Availability

No datasets were generated or analysed during the current study.

## Abbreviations

2D-STE: Two-dimensional speckle tracking echocardiography
ASE/EACVI: American Society of Echocardiography and European Association of Cardiovascular Imaging
CAD: Coronary artery disease
CCC: Cirrhotic Cardiomyopathy Consortium
CCM: Cirrhotic cardiomyopathy
CO: Cardiac output
DSE: Dobutamine stress echocardiography
ECG: Electrocardiogram
ESLD: End-stage liver disease
GLS: Global longitudinal strain
HPS: Hepatopulmonary syndrome
HVPG: Hepatic venous pressure
IPVD: Intra-pulmonary vascular dilation
LT: Liver transplantation
LV: Left ventricular
LVEF: Left ventricular ejection fraction
LVOTO: Left ventricular outflow tract obstruction
MELD: Model of end-stage liver disease
mPAP: Mean pulmonary artery pressure
NASH: Non-alcoholic steatohepatitis
PAH: Pulmonary arterial hypertension
PAP: Pulmonary artery pressure
PoPH: Portopulmonary hypertension
PVR: Pulmonary vascular resistance
RAAS: Renin-angiotensin-aldosterone system
RAP: Right atrial pressure
RHC: Right heart catheterization
RVSP: Right ventricular systolic pressure
SVR: Systemic vascular resistance
TDI: Tissue Doppler imaging
TIPS: Transjugular intrahepatic portosystemic shunt
TRV: Tricuspid regurgitant velocity
TTCE: Transthoracic contrast echocardiography
TTE: Transthoracic echocardiography
